# Neuropathy in ARSACS is demyelinating but without typical nerve enlargement in nerve ultrasound

**DOI:** 10.1007/s00415-023-12159-2

**Published:** 2024-01-23

**Authors:** Katharina Kneer, Stephanie Straub, Julia Wittlinger, Jan-Hendrik Stahl, Natalie Winter, Dagmar Timmann, Ludger Schöls, Matthis Synofzik, Friedemann Bender, Alexander Grimm

**Affiliations:** 1 Department of Epileptology, Center of Neurology, Universitätsklinikum Tübingen, Hoppe-Seyler-Str. 3, 72076 Tübingen, Germany; 2 Hertie Institute for Clinical Brain Research, Eberhard-Karls University Tübingen, Tübingen, Germany; 3 Department of Neurodegenerative Diseases, Center of Neurology, University of Tuebingen, Tuebingen, Germany; 4 Department of Neurology and Center for Translational Neuro- and Behavioral Sciences (C-TNBS), Essen University Hospital, University of Duisburg-Essen, Essen, Germany; 5 German Center for Neurodegenerative Diseases (DZNE), Tuebingen, Germany; 6 Kinder- Und Jugend Psychiatrie Klink Esslingen, Esslingen, Germany

**Keywords:** Ataxia, ARSACS, Neuropathy, Ultrasound, UPSS

## Abstract

**Background:**

To specify peripheral nerve affection in autosomal recessive spastic ataxia of Charlevoix-Saguenay (ARSACS) by correlating high-resolution nerve ultrasound and nerve conduction studies.

**Methods:**

We assessed a cohort of 11 ARSACS patients with standardized nerve conduction studies and high-resolution ultrasound of peripheral nerves and compared nerve ultrasound findings to a healthy control group matched for age, sex, size and weight.

**Results:**

Mean age of patients was 39.0 (± 14.1) years and disease duration at assessment 30.6 (± 12.5) years. All patients presented with a spasticity, ataxia and peripheral neuropathy. Neuropathy appeared to be primarily demyelinating in 9/11 cases and was not classifiable in 2/11 cases due to not evocable potentials. Nerve ultrasound revealed a normal ultrasound pattern sum score (UPSS) in each ARSACS patient and no significant nerve enlargement compared to the control group.

**Conclusions:**

Peripheral neuropathy in ARSACS showed primarily demyelinating rather than axonal characteristics and presented without nerve enlargement. As demyelinating neuropathies do commonly present enlarged nerves we recommend further genetic testing of the SACS gene in patients who present with this combination of demyelinating neuropathy without nerve enlargement. ARSACS cases that initially presented only with neuropathy without spasticity or ataxia and therefore were misdiagnosed as Charcot-Marie-Tooth disease are supporting this suggestion.

## Introduction

High-resolution ultrasound (HRUS) of nerves is a rapidly expanding field. As an addition to nerve conduction studies (NCS) it helps further stratify neuropathies.

Generally, ultrasound examinations show nerve enlargement in demyelinating neuropathies, whereas most axonal neuropathies do not [1]. Previous data show nerve enlargement in hereditary caused neuropathies like Charcot–Marie–Tooth disease (CMT) [2–5] or hereditary neuropathy with liability to pressure palsy (HNPP) [4, 5]. Nerve enlargement was also reported for autoimmune caused neuropathies like chronic inflammatory demyelinating polyneuropathy (CIDP) [2, 3, 6], Guillain–Barre Syndrome (GBS) [7], multifocal motor neuropathy (MMN) [2, 8], demyelinating neuropathy in monoclonal gammopathy of undetermined significance (MGUS) [9], and neuropathy with anti-MAG (myelin-associated glycoprotein) antibodies [10].

In contrast, axonal neuropathies commonly do not show nerve enlargement or only to a mild degree. Axonal neuropathy caused by vasculitis [11, 12], sarcoidosis [13], or sporadic mononeuropathies without trauma (e.g., borreliosis or hepatitis E) [14] are exceptions that often present with focal nerve swelling.

There is a lack of data regarding HRUS in genetic neurodegenerative diseases other than primary hereditary neuropathies. Recently nerve enlargement was shown in demyelinating X-linked adrenomyeloneuropathy (AMN) [15], metachromatic leukodystrophy [16], and cerebrotendinous xanthomatosis (CTX) [17].

This study investigates HRUS data compared to electrophysiological data in patients suffering from autosomal recessive spastic ataxia of Charlevoix-Saguenay (ARSACS).

ARSACS is caused by bi-allelic mutations in the SACS gene encoding sacsin and is characterized as an early onset spastic cerebellar ataxia with peripheral neuropathy [18]. Characteristic but not obligatory imaging findings include streaky pontine hypointensities in magnetic resonance imaging (MRI) and in some cases also hypertrophic retinal myelinated fibers. Further, epilepsy, hearing loss, urinary dysfunction, and mild intellectual disability have been reported [19–24]. Cases have been reported, which primarily presented with an isolated neuropathy, leading to the initial diagnosis of CMT [19, 25].

Findings in nerve conduction studies (NCS) in these patients are heterogenic and include not only primarily axonal polyneuropathic but also primary demyelinating or mixed demyelinating and axonal changes [19, 21–23, 26].

## Patients and methods

### Cohort

Included in this study were 11 genetically diagnosed ARSACS patients with homozygous or two compound heterozygous mutations in the SACS gene (Table 1).

**Table 1:** Study cohort

	A01	A02	A03	A04	A05	A06	A07	A08	A09	A10	A11
Sex	f	m	f	f	f	m	m	m	m	m	m
Age [y]	53	33	45	52	53	23	33	36	13	31	57
Disease duration [y]	26	30	39	51	51	23	33	22	12	31	18
Mutation	R961*/1586_1587del	R728*/M3551Nfs*4	R728*/F4357Lfs*11	L3379*/Y3759del+I3758M	Q1652*/Q1709*	L3379*/F3027Lfs*25	T2388Rfs*10/T2388Rfs*10	R1575W/R1575W	R2426P/Y2975Ffs*29	M1359R/M1359R	Q1709*/L3156F
ARSACS related signs/symptoms											
Cerebellar ataxia	✓	✓	✓	✓	✓	✓	✓	✓	✓	✓	✓
Pyramidal symptoms /spasticity	✓	✓	✓	✓	✓	✓	✓	✓	✓	✓	✓
Peripheral neuropathy	Demyel.	Demyel.	Demyel.	Demyel	Demyel.	NA	Demyel.	Demyel.	Demyel.	Demyel.	Demyel.
Paresis	✓	✓	✓	✓	✓	✓	✓	/	✓	✓	✓
Dystonia	/	/	facial	/	/	/	/	/	/	/	/
Dysarthria	✓	✓	✓	✓	✓	✓	✓	✓	✓	✓	/
Dysphagia	✓	✓	✓	✓	✓	✓	/	✓	/	✓	/
Nystagmus	✓	✓	✓	✓	✓	✓	✓	✓	✓	✓	✓
Cognitive impairment	/	/	/	✓	✓	/	/	/	/	/	/
Epilepsy/history	/	✓	/	✓	✓	✓	/	/	/	/	/
Urinary dysfunction	✓	✓	✓	✓	✓	/	✓	✓	/	/	✓
Hypacusis	✓	/	/	/	/	/	/	/	/	/	/
Further symptoms or diagnoses	Depression, dilative CMP, diabetes mellitus type 2	/	/	Cataract, panic attacks	/	/	/	/	/	/	Depression, migraine, hepatic steatosis
SARA	26	23.5	26	24	29.5	12	16.5	14	12	23	8
NCS											
Motor	U, F, T	U, F, T	U, F, T	U, F, T	U, F, T	F, T	U, F, T	U, F, T	U, F, T	U, F, T	U, F, T
Sensory	U, S	U, S	U, S	U, S	U, S	S	U, S	U, S	U, S	U, S	U, S
UPSS	✓	✓	✓	✓	✓	✓	✓	/	✓	✓	✓

*f* female, *m* male, *demye* demyelinating polyneuropathy, *SARA* scale for the assessment and rating of ataxia, *NCS* nerve conduction study, *UPSS* ultrasound pattern sum score, *Ul* ulnar nerve, *fl* fibular nerve, *Tr* tibial nerve, *S* sural nerve

Between 09/2019 and 06/2020 the participants have been examined in our specialized outpatient clinic for ataxia and received an additional electrophysiological work-up, including NCS and nerve ultrasound. This study was approved by the local ethic committee of the University of Tübingen (702/2015BO2) and conform with the World Medical Association Declaration of Helsinki. All examinations were undertaken with understanding and written informed consent from all participants. Further nerve ultrasound measurements were obtained for an age, sex, size, and weight-matched healthy control group with no medical history or symptoms of neuropathy.

### Clinical assessment and electrophysiology

The clinical assessment involved a standardized neurological examination by a movement disorder specialist, including the Scale for the Assessment and Rating of Ataxia (SARA) [27].

Nerve conduction studies were performed with standard conditions as described before [28], using a Dantec^®^ Keypoint^®^ G4 workstation (Natus Medical Inc., San Carlos, California, USA). Motor nerve conduction studies included distal motor latencies (DML), compound muscle action potentials (CMAP), and motor nerve conduction velocities (MNCV) of the ulnar (U), fibular (F), and tibial nerve (T). Sensory NCS included sensory nerve action potentials (SNAP) and sensory nerve conduction velocities (SNCV) of the ulnar (U) and sural nerve (S).

In all patients, motor nerves were tested alternately on both sides, for example, left side for ulnar and tibial nerve, right side for the fibular nerve. Sensory nerves were tested on the right side; however, changes were made individually to each patient, without deviating from the overall protocol.

Neuropathy was defined as demyelinating if NCV was slower than 75% or distal latency longer than 130% of the norm and as axonal if amplitudes were decreased with only mild reduction of NCV or distal latency, accordingly to Preston and Shapiro 2013 [29] (comparable to the guidelines of the European Federation of Neurological Societies and the Peripheral Nerve Society for CIDP [30]).

### High-resolution nerve ultrasound

High-resolution B-mode ultrasound was performed accordingly to a previously published protocol [17] with a high-resolution probe (24 MHz broad band linear probe, Aplio i800, Canon Medical Systems GmbH, Neuss, Germany). Nerve ultrasound covered easily accessible peripheral nerves on the right body site (median, ulnar, radial, tibial, fibular, sural and vagal nerve, as well as the C5 and C6 nerve roots) and was performed by well-experienced sonographers, assessing the cross-sectional area (CSA) of peripheral nerves and diameter for cervical roots. In a nerve enlargement of > 150% 2 points and in a nerve enlargement > 100% but less than 150% 1 point are given.

With adjusted boundary values we summed up the results to the Ultrasound Pattern Sum Score (UPSS) reaching from 0 to 22 points, whereas a sum of ≤ 3 points is supposed to be not pathological [31]. For A09 adolescent boundary values were used [32].

Echo intensity was evaluated semi-quantitatively by defining nerves as hypoechogenic if isointense with blood vessels lumen and hyperechogenic if isointense with lymph nodes [6].

### Statistical analysis

All analyses were conducted using IBM SPSS Statistics, version 27.0.0.0 (Chicago, IL, USA). Results of descriptive analyses are given as mean ± standard deviation (SD) if normally distributed or median and inter quartile range (IQR) if not normally distributed, after testing normality with the Shapiro–Wilk test. ARSACS patients and the control group were compared using an independent-sample *t*-test for normally distributed variables (with testing the equality of variance by the Levene test) and for non-normally distributed variables with the Mann–Whitney *U* test. Sex, as the only categorical variable, was not tested because of exact matching in both groups. Significance level for the nerve ultrasound results was corrected for multiple comparisons and set at *p* ≤ 0.003. For all other tests significance level was set at *p* ≤ 0.05.

## Results

### Cohort

An overview of the included participants with their clinical and genetic characteristics is given in Table 1. The mean age at assessment was 39.0 (± 14.1) years with a mean disease duration of 30.6 (± 12.5) years since retrospectively the first ARSACS-related symptom occurred.

### Clinical assessment and electrophysiology

In total, 11 patients, registered to our outpatient ataxia department of the university hospital Tuebingen and Essen diagnosed with ARSACS, were included in this study. All patients clinically presented with the typical triad of ARSACS including spasticity, ataxia, and peripheral neuropathy (see Table 1). Mean SARA score was 19.5 (± 7.2) points.

In all 11 cases SNAP of the sural and ulnar nerve was extinguished.

NCS showed a demyelinating neuropathy in 10/11 cases, defined by a reduced NCV of ≤ 75% of the norm or a distal latency longer than 130% of the norm. In one patient, neither MSAP nor SNAP could be evoked; therefore, no further classification was possible. Late response was determined for the ulnar and tibial nerve. Since the MUAP amplitude of the tibial nerve was not evocable in 8 of 11 patients, the late response of the ulnar nerve was primarily considered when conducting the analysis. In 5 of 8 patients’ late response was elongated by > 30% (see Table 2). All nerve conduction study data values were compared to normative values adapted from literature.

**Table 2:** Individual electrophysiological and nerve ultrasound findings

	A01	A02	A03	A04	A05	A06	A07	A08	A09	A10	A11	Norm
HRUS												
Median nerve [mm^2^]	8/9/8	10/8/3	7/11/5	6/6/5	9/9/6	9/8/4	7/8/4	NA	12/9/5	9/9/4	10/8/7	< 12/12/10^a^
Ulnar nerve [mm^2^]	6/6/5	5/5/3	6/7/4	5/4/4	5/6/4	6/3/4	5/8/5	NA	7/5/4	7/6/5	8/8/6	< 9.5/10/8.5^a^
Radial nerve [mm^2^]	1	1	1	1	1	1	1	NA	1	1	1	< 3^a^
Tibial nerve [mm^2^]	17/31	13/32	9/17	19/29	7/31	8/21	7/26	NA	8/19	13/32	14/52	< 14/33^a^
Fibular nerve [mm^2^]	1/11	3/6	1/5	2/7	2/9	1/5	2/9	NA	2/6	1/7	2/11	< 3.5/11.5^a^
Sural nerve [mm^2^]	1	2	3	3	3	2	1	NA	3	2	1	< 3.5^a^
Vagal nerve [mm^2^]	3	2	3	2	3	2	1	NA	2	2	1	< 3.5^a^
C5/C6 [mm]	2.3/2.1	2.7/4.0	2.3/3.9	2.5/4.6	2.7/3.7	2.4/4.1	2.8/4.7	NA	2.6/3.5	2.8/3.8	2.6/4.0	< 2.9/4.2^a^
UPSA	1	0	0	1	0	0	0	NA	2	0	2	
UPSB	0	0	0	1	0	0	1	NA	0	0	0	
UPSC	0	0	0	0	0	0	0	NA	0	0	0	
UPSS	1	0	0	2	0	0	1	NA	2	0	2	< 3
NCS												
DML [ms]												
Ulnar nerve	4.4	4.4	4.9	2.9	2.7	NA	3.4	3.2	3.5	4.8	3.1	≤ 3.2
Fibular nerve	–	–	–	–	–	–	6.9	6.5	–	–	–	≤ 4.0
Tibial nerve	–	6.4	–	–	–	–	–	6.4	5.0	–	–	≤ 5.1
CMAP [mV]												
Ulnar nerve	7.3/5.7	11.2/9.2	9.5/6.2	6.9/4.7	13.0/12.0	NA	10.4/8.9	13.9/12.1	10.1/10.5	6.4/5.8	13.5/10.9	≥ 3.2
Fibular nerve	–	–	–	–	–	–	2.5/2.3	0.7/0.7	–	–	–	≥ 4.0
Tibial nerve	–	2.7/2.6	–	–	–	–	–	4.5/2.2	3.0/2.9	–	–	≥ 5.1
MNCV [m/s]												
Ulnar nerve	34	43	40	49	36	NA	53	41	43	39	55	≥ 50
Fibular nerve	–	–	–	–	–	–	27	26	–	–	–	≥ 41
Tibial nerve	–	29	–	–	–	–	–	34	31	–	–	≥ 40
SNAP [mV]												
Ulnar nerve	–	–	–	–	–	NA	–	–	3.0	–	2.6	≥ 5.8
Sural nerve	–	5.9	–	–	–	–	–	–	4.3	–	–	≥ 3.8
SNCV [m/s]												
Ulnar nerve	–	–	–	–	–	NA	–	–	49	–	44	≥ 44
Sural nerve	–	36	–	–	–	–	–	–	42	–	–	≥ 39

Individual findings of nerve conduction studies and high-resolution nerve ultrasound

Ultrasound results for nerves are given as CSA (in mm^2^, from proximal to distal) and for the cervical nerve roots as diameter (in mm) [31]

*C5/C6* cervical root 5 and 6, *UPSA* part A of the UPSS, *UPSB* part B of the UPSS, *UPSC* part C of the UPSS, *NA* not available

^a^Standard values for adults. For A09 standard values are differing according to a 13 years old as described in Grimm et al. [32]

### High-resolution nerve ultrasound

**Fig. 1:**
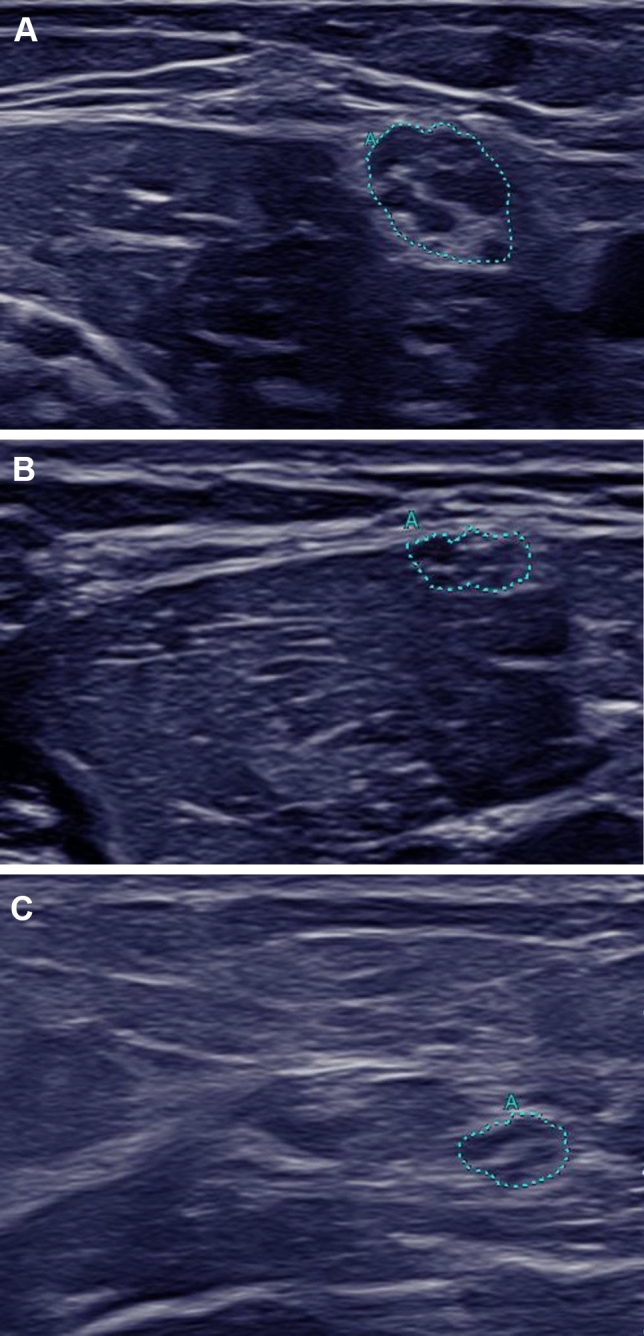
Nerve ultrasound of the right ulnar nerve measured at the upper arm

Nerve ultrasound was performed in 10 of 11 patients, in 5 investigated patients subtle nerve swelling was seen. The UPSS varied from a minimum of 0 to 2 points and is therefore non-pathological (Tables 2 and 3). Exemplary ultrasound images of the right ulnar nerve at the upper arm are shown in Fig. 1 for one ARSACS patient (A01) in comparison to a CIDP patient and a control group patient. The distribution pattern was inhomogenic and nerve swelling did not only affect the peripheral nerves but also affected the cervical nerve roots. Nerve ultrasound results were compared to a control group with no significant difference in sex, age, size, and weight. After correcting for multiple testing (*p* ≤ 0.003), no significant differences of CSA values were observed. The results of the group comparison are reported in Table 3.

**Table 3:** Comparison of nerve ultrasound results between ARSACS and healthy controls group

	ARSACS (*n* = 10)			Controls (*n* = 10)			*p* values	
Sex	4 f/6 m			4 f/6 m				
	Mean (± SD)	Median (IQR)	Range	Mean (± SD)	Median (IQR)	Range	*t* test	Mann–Whitney *U*
Age [y]	39.3 (± 14.9)	39.0 (24.0)	13–57	42.2 (± 12.5)	41.5 (22.8)	25–60	0.643	
Size [cm]	170.9 (± 9.8)	172.0 (19.8)	156–183	172.6 (± 6.4)	172.0 (13.3)	163–183	0.650	
Weight [kg]	79.0 (± 15.7)	76.5 (27.8)	61–102	70.4 (± 8.3)	72.0 (11.3)	56–80	0.149	
Median nerve [CSA in mm^2^]								
Upper arm	8.7 (± 1.8)	9.0 (3.0)	6–12	8.6 (± 1.6)	9.0 (3.0)	6–11	0.895	
Cubital	8.5 (± 1.3)	8.5 (1.0)	6–11	8.9 (± 1.5)	9.0 (2.5)	7–11	0.520	
Forearm	5.4 (± 1.8)	5.0 (3.3)	3–8	6.6 (± 1.4)	6.5 (3.0)	5–8		0.105
Ulnar nerve [CSA in mm^2^]								
Upper arm	6.0 (± 1.1)	6.0 (2.0)	5–8	6.8 (± 1.0)	6.5 (1.3)	6–9		0.123
Cubital	5.8 (± 1.6)	6.0 (2.5)	3–8	8.1 (± 1.5)	8.5 (1.5)	5–10	0.004	
Forearm	4.4 (± 0.8)	4.0 (1.0)	3–6	5.6 (± 1.3)	5.5 (1.5)	4–8	0.022	
Radial nerve [CSA in mm^2^]								
Superficialis	1.0 (± 0.0)	1.0 (0.0)	1–1	1.8 (± 0.6)	2.0 (1.0)	1–2		0.007
Tibial nerve [CSA in mm^2^]								
Popliteal	11.5 (± 4.3)	11.0 (7.0)	7–19	9.9 (± 2.4)	9.0 (3.5)	8–15		0.739
Malleolar	29.0 (± 9.8)	30.0 (11.5)	17–52	22.3 (± 4.8)	24.0 (8.5)	14–28	0.069	
Fibular nerve [CSA in mm^2^]								
Popliteal	7.6 (± 2.3)	7.0 (3.8)	5–11	8.2 (± 1.0)	8.0 (2.0)	7–10	0.461	
Superficialis	1.7 (± 0.7)	2.0 (1.0)	1–3	2.1 (± 0.6)	2.0 (0.3)	1–3		0.218
Sural nerve [CSA in mm^2^]								
Lower leg	2.1 (± 0.9)	2.0 (2.0)	1–3	2.0 (± 0.5)	2.0 (0.0)	1–3		0.739
Vagal nerve [CSA in mm^2^]								
Carotid sheath	2.1 (± 0.7)	2.0 (1.3)	1–3	2.1 (± 0.6)	2.0 (0.3)	1–3		0.971
Cervical roots [diameter in mm]								
C5	2.6 (± 0.2)	2.6 (0.4)	2.3–2.8	2.5 (± 0.2)	2.5 (0.4)	2.1–2.7	0.327	
C6	3.8 (± 0.7)	4.0 (0.6)	2.1–4.7	3.6 (± 0.4)	3.6 (0.6)	2.9–4.4		0.063

Further, nerve ultrasound of an exemplary patient suffering from chronic inflammatory demyelinating polyneuropathy (CIDP) showed a statistically significant higher UPSS (*p* < 0.0.5).

## Discussion

Due to the rarity ARSACS, the current literature lacks a clear description of NCS findings and ultrasound data. Especially in regard to NCS, data described in literature are contradictive.

In the previously described studies, demyelinating or axonal neuropathy was either not clearly defined or defined as demyelinating if nerve conduction velocity was reduced, axonal if action potentials were affected, or mixed if both signs were seen, while the current EAN or AAN criteria and guidelines were not taken into consideration.

A further limitation is the ambiguity of which nerves were examined. Therefore, specified data vary from a clear description of which nerves are included in the performed NCS [19, 20, 22, 26], while other studies do not provide any further details [21, 23]. Our data show that ARSACS patients present with demyelinating polyneuropathy, accompanying the underlying pathophysiology of the disease:

To date, the role of sacsin is not fully understood, although an axonal and Schwann cell dysfunction in patients with sacsin mutations is implicated [33].

Therefore, ARSACS differs from other common recessive ataxias with neuropathy like polymerase gamma (POLG)-related ataxia [34, 35], the cerebellar ataxia, neuropathy and vestibular areflexia syndrome (CANVAS) [36], Friedreich’s ataxia (FRDA) [37], Ataxia telangiectasia (AT) [38], and ataxia with oculomotor apraxia (AOA) Type 1 [39] and 2 [40], which are all presenting a mainly axonal type of neuropathy. Histopathologically, peripheral nerve findings in ARSACS are well reflecting demyelination by demonstrating onion bulbs, thinning of myelin sheaths, and loss of large myelinated fibers, but also show signs of axonal damage [20, 26, 41, 42].

Nerve ultrasound showed no nerve enlargement in all patients with an UPSS of < 3 points which in turn is contradictory to our previous understanding of demyelinating polyneuropathies:

Generally speaking, nerve swelling can be seen on ultrasound, although with some individual differences. Inflammatory polyneuropathies, such as CIDP and atypical CIDPs, tend to show focal nerve swelling with some predominant fascicles [2, 3, 6]. Hereditary polyneuropathies, such as Charcot–Marie–Tooth Type 1 or storage diseases like leukodystrophies, adrenoleukodystrophies, xantochromatosis, or glucocerebrosidosis [15–17, 32, 43], mainly show ubiquitous nerve swelling, as would be expected in ARSACS patients per se. Our knowledge on nerve ultrasound as a biomarker in neurodegenerative diseases still lacks data. So far studies were able to identify that no nerve swelling is observed in other neurodegenerative diseases, spinocerebellar ataxia type 2 (SCA2), and CANVAS [44] or spinal muscular atrophy (SMA) [45]. However, as mentioned previously, these patients present with predominant axonal damage on NCS and ultrasound data in these cases coincide with ultrasound data from other, non-genetic, axonal neuropathies, making ARSACS in total, an exemption.

High-resolution ultrasound itself serves as an important biomarker to further stratify polyneuropathies as a non-invasive and fast diagnostic tool. We therefore suggest that in patients primaprily presenting with polyneuropathy, not ataxia, combined with primarily demyelinating peripheral neuropathy and no nerve swelling seen on ultrasound, further genetic testing for ARSACS should be strongly considered.

Early diagnosis prevents patients from vain diagnostic procedures and enables adequate counseling and symptomatic treatment. ARSACS patients who have been initially misdiagnosed as CMT support this assumption [19, 25].

A limitation of our study is its small sample size. If possible in this ultra-rare disorder, additional studies with larger patient numbers and extended electrophysiological examinations, nerve ultrasound, and correlation with histopathological data are needed to further evaluate the reported results. A correlation between neuropathy severity and ataxia severity has also been not assessed in this study design, but should be assessed in other prospective study designs.

## Conclusion

In summary, this high-resolution ultrasound and electrophysiological study indicates a primarily demyelinating neuropathy pattern in ARSACS without enlargement of peripheral nerves. We therefore recommend genetic testing for mutations in the SACS gene in patients with demyelinating neuropathy who are not presenting enlargement of peripheral nerves.

## Data Availability

The data that support the findings of this study are available from the corresponding author upon request.
